# Symptoms Indicating Imminent Breakage of a Femoral Interlocking Nail: A Case Report

**DOI:** 10.5704/MOJ.1311.003

**Published:** 2013-11

**Authors:** KL Pan, WH Chan

**Affiliations:** Department of Orthopaedics, Universiti Malaysia Sarawak, Kuching, Malaysia; Department of Orthopaedics, Universiti Malaysia Sarawak, Kuching, Malaysia

## Abstract

Fractures of the femoral shaft treated with interlocking nails will ultimately result in breakage of the nail if the bone does not unite. Further management requires removal of the broken nail which may be a difficult process for the distal segment. If we can identify the symptoms just before the nail breaks, an exchange nailing becomes much easier. We present a patient with fibromatosis who underwent repeated surgery as well as radiotherapy at the age of 16. Six years later, she had a pathological fracture of the upper third of the femur for which an interlocking nail was inserted. The femur did not unite and the nail subsequently broke. Over a period of 12 years, three nails broke and had to be replaced. Two to 3 months before each breakage, the patient experienced the same set of symptoms for each episode. Knowing that her fracture was not going to heal will now alert us to do an exchange nailing before the nail broke again. It is well known that where there is evidence of non-union, pre-emptive treatment is necessary before implant failure.

**Key Words:**

Femur nonunion, interlocking nail, symptoms before breakage of nail

## Introduction

Fractures of the femoral shaft are often treated with locked intramedullary nails, with good results. However, there are instances when the fracture fails to unite^1^. This is especially so when radiotherapy had previously been given^2^,^3^.

When the bone does not unite, stress is taken by the nail during weight-bearing. Over a certain period of time, the nail will fail and break. When this happens, the broken nail has to be removed and a new one put in place, sometimes supplemented with bone grafting. After a nail has broken, it is often difficult to remove the distal segment^1^.

If it is known when the nail is about to break (imminent) an exchange nailing prior to the breakage is usually a straightforward procedure. We present a patient who had four nail breakages over a period of 12 years. The femur fracture failed to unite because radiotherapy had been given. We elucidate the symptoms that she experienced before each breakage, which was found to be similar on each occasion.

## Case Report

**Fig. 1:**
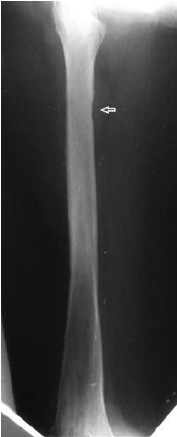
: Plain x-rays of the femur after radiotherapy in 1998.Arrow shows an area of cortical thickening which would predispose to future fracture.

A 34-year old female was first diagnosed with fibromatosis of the right thigh in 1994 when she was 16 years old, for which an excision was done. It recurred two years later. After a reexcision, there was some residual tumour and 25 fractions of radiotherapy was given. A year later, the swelling recurred again and an excision was done for the third time. It recurred for a fourth time 2 years later and this time, another 25 fractions of radiotherapy was given without surgical excision. (Figure 1)

A year after the last radiotherapy treatment (6 years after presentation) in 2000, the femur fractured spontaneously at the shaft (Figure 2a) and this was treated with a Targon interlocking nail. (Figure 2b) Bone grafting was done when it had not united 20 months later. (Figure 2c) Periodic followup did not document union. In 2007, seven years after the nail was inserted, it broke at the nonunion site. Three months prior to the breakage, she experienced needling, intermittent pain (like ant bites) at the nonunion site. It was worse at night and on rainy days, and also after prolonged standing and walking. This progressively worsened until a few days before breakage when it became most severe on standing up from a sitting position. At the point of breakage, there was a sudden, severe pain and inability to bear weight, followed by swelling and warmth over the thigh. The patient was then brought to the hospital where an radiograph confirmed a broken nail. An exchange nailing was done a few days later.

A year later, the nail broke again. (Figure 3a) She had gained weight during this time and had been walking a lot at a new job. An exchange nailing was duly done. This third nail broke 4 years later in 2012 and was followed by the third exchange nailing. (Figure 3b)

Two to three months before each breakage, the same episode of pain was repeated as detailed previously. She was even able to warn the attending doctor that the nail was about to break.However, without proof of breakage on radiographs, nothing was done.

At the time of the actual breakage, there would be sudden severe pain followed by swelling of the thigh and complete inability to bear weight.

## Discussion

When there is established nonunion in a femoral shaft fracture previously treated by an intramedullary nail, the treatment is usually a straightforward exchange nailing with reaming and replacing the nail with a larger diameter nail. The original nail is easily removed and a new nail is reinserted through the same opening and tract. However, when the nail is also broken at the nonunion site, removal of the distal segment often becomes technically difficult^1^. Sometimes, it necessitates the opening up of the fracture site with removal of more bone and callus before the distal end can be extracted.

In our patient, a large dose of radiotherapy had been given and union was expected to be a problem. Three exchange nailings had been done together with one accompaniment of bone grafting on one occasion. Each time, the nail had broken extraction of the distal segment was a tricky procedure. On closer scrutiny of the patient’s history, we found that the same symptomatology had preceded each episode of nail breakage. Two to three months before each breakage, she would begin experiencing intermittent, needling pain (like ant bites). This pain was worse with lower ambient temperatures (at night and on rainy days) and on prolonged standing and walking. These symptoms would progress and culminate in a severe pain on standing up from a sitting position. This would be the “imminent stage” and the nail would break a few days later. When it actually breaks, the patient is instantly aware of it by the inability to bear weight, swelling and warmth.

On a search of the literature, we found a similar case report of fatigue failure of an AO spiral blade for a femoral fracture which only became radiologically visible 4 months after the start of the symptom of pain^4^.

The human body is a harsh environment for implanted metallic materials and even the most corrosion resistant material undergo chemical or electrochemical dissolution. Our patient’s Targon implant is made of titanium, which forms a stable TiO2 film which releases titanium particles under wear into the body environment^5^.

It is our belief that the chemical and particle release into the surrounding tissue becomes maximal just before the implant breaks causing the characteristic symptoms in this patient. The reported incidence of nail failure is between 1.7% and 5.6%^1^. If it is known that the implant is about to fail, exchange nailing couls be done before the breakage occurs, obviating the possibility of a difficult extraction. Further studies will need to be carried out to see if this can be extrapolated to the larger population of traumatic femoral fractures treated with locking nails which do not unite.

**Fig. 2a:**
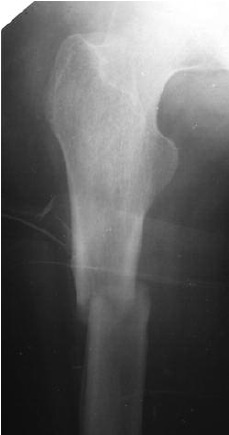
: Plain x-rays of the fracture in 2000.

**Fig. 2b:**
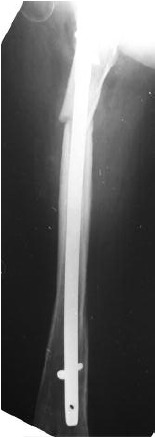
: Interlocking nail done after the fracture in 2000.

**Fig. 2c:**
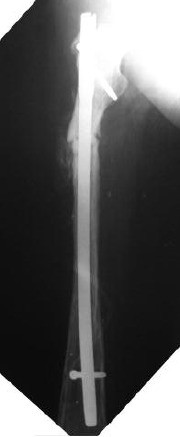
: Bone grafting done in 2002 when the bone did not unite.

**Fig. 3a:**
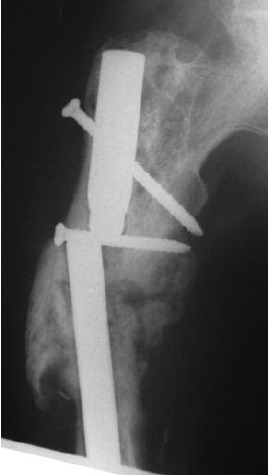
: Nail breakage in 2008.

**Fig. 3b:**
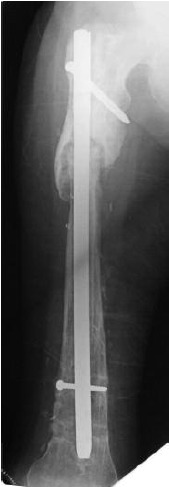
: The last interlocking nail inserted in 2012. The fracture site has not united.
